# A case of safe airway management by fiber-optic nasotracheal intubation in general anesthesia in a pediatric patient with Hajdu-Cheney syndrome: a case report

**DOI:** 10.1186/s40981-023-00627-7

**Published:** 2023-06-12

**Authors:** Atsushi Kokita, Tomohiro Chaki, Michiaki Yamakage

**Affiliations:** Department of Anesthesiology, Sapporo Medical University School of Medicine, South 1, West 16, Chuo-Ku, Sapporo, Hokkaido 060-8543 Japan

**Keywords:** Hajdu-Cheney syndrome, Difficult airway, Nasotracheal intubation, Airway management

## Abstract

**Background:**

Hajdu-Cheney syndrome (HCS) is an extremely rare disorder characterized by progressive acro-osteolysis. A unique facial structure and deformity of the cervical spine are associated with a difficult airway. Although several reports describe general anesthesia with orotracheal intubation for patients with HCS, there have been no reports of nasotracheal intubation with a risk of skull base fracture. We describe nasotracheal intubation for oral surgery in a patient with HCS.

**Case presentation:**

A 13-year-old girl with HCS was scheduled for dental surgery. Preoperative computed tomography revealed no abnormalities including fractures in the skull base or cervical spine. After confirming a lack of vocal cord paralysis by bronchofiberscopic inspection from the nose, general anesthesia was induced with sevoflurane, remifentanil, and rocuronium. Fiber-optic nasotracheal intubation was successfully performed without complications such as depletion of oxygen saturation and massive epistaxis, and the surgery was completed uneventfully. She was discharged the day after surgery with no anesthesia-related complications.

**Conclusions:**

We were able to safely manage the airway of a patient with HCS by nasotracheal intubation under general anesthesia.

## Background

Hajdu-Cheney syndrome is a rare, autosomal dominant disorder comprising acro-osteolysis of the distal phalanges with associated digital abnormalities, distinctive craniofacial and skull changes, dental anomalies, and proportionate short stature [1]. An important neurological issue is platybasia, which may cause compression of the brain stem, the cranial nerve, and the cerebellum. Obstructive sleep apnea, vocal cord paralysis, dysarthria, and dysphagia have also been reported in patients with HCS [2]. Although there have been a few reports on general anesthesia in patients with HCS, there has been no reports on airway management by nasotracheal intubation during general anesthesia in a patient with HCS. Previously reported cases were managed by orotracheal intubation because of the risk of fracture at the skull base [2, 3]. We report a case in which nasotracheal intubation was successfully performed to manage the airway of a patient with HCS.

## Case presentation

Approval and written informed consent for publication of this report were obtained from the patient and her mother. The manuscript adheres to the CAse REport (CARE) guidelines [4, 5].

The patient was a 13-year-old girl who weighed 32.7 kg and was 141.7 cm in height. She was born with a low birth weight. She was initially suspected to have Down syndrome due to her characteristic facial features and atrial septal defect (ASD), but chromosome testing revealed a normal karyotype. She attended a regular elementary school for 6 years. When she was in the seventh grade, genetic testing was performed and revealed a mutation in the NOTCH2 gene, leading to a definitive diagnosis of HCS. No other family members had this syndrome. Her psychomotor development was normal. She had a medical history of well-controlled asthma and bilateral conductive hearing loss and osteoporosis. She underwent a total of six surgeries for congenital hip dislocation under general anesthesia between the ages of 0 and 2 years. In previous surgeries, she was orally intubated with a Macintosh laryngoscope, and her airway was managed without any problems.

She had been concerned about the alignment of her front teeth for several years. She was referred to our oral surgery department from our genetic medicine department for evaluation of her orthodontic needs. Since an examination revealed residual deciduous teeth and retarded eruption of permanent teeth, she was scheduled for extraction of deciduous teeth and mucous membrane incisions in the crowns of the teeth. Preoperative airway assessment revealed that cervical retroflection was normal, and the Mallampati score was class II, but the interincisal distance was 3 cm, and the thyromental distance was 6 cm, and one canine tooth was upset. Airway assessment using the Arne risk index showed a simplified score of 19, indicating that tracheal intubation would be difficult [6]. We performed preoperative computed tomography (CT) scans of the skull base and cervical spine to ensure that there were no anatomic abnormalities such as skull base fractures or cervical compression fractures. Preoperative examinations including a blood test, electrocardiogram, and chest X-ray showed no significant findings. She had exercise tolerance of more than 4 metabolic equivalents.

We planned general anesthesia with fiber-optic nasotracheal intubation. The patient entered the operating room calmly without premedication. After starting standard monitoring and inserting a venous catheter, we confirmed the absence of vocal cord paralysis with a transnasal bronchofiberscope. General anesthesia was induced with remifentanil 0.3 µg/kg/min, propofol 3 mg/kg, and rocuronium 0.6 mg/kg under monitoring of bispectral index. After induction of general anesthesia, mask ventilation was easily performed by two anesthesiologists in a cervical midline immobilized position. To avoid skull base fracture due to nasal airway insertion, a 12 Fr suction catheter was first inserted into her nasal cavity, and a nasal airway (internal diameter of 5.5 mm) was carefully inserted using a suction catheter as a guide. Fiber-optic nasotracheal intubation was smoothly performed with her neck fixed in the midline position and the mouth not open. The airway management was completed without complications such as depletion of oxygen saturation and massive epistaxis. For anesthesia maintenance, total intravenous anesthesia (TIVA) with continuous administration of propofol at 8–10 mg/kg/h and remifentanil at 0.2–0.3 µg/kg/min was used for maintaining a target BIS of 40–60 during surgery. The surgeon administered infiltration anesthesia with 4 mL of 2% lidocaine before the surgery. We also administered 15 mg/kg of acetaminophen just before the end of the surgery to reduce the patient’s postoperative pain. The surgery was completed without problems; the operation time was 1 h and 3 min, and the anesthesia time was 2 h and 29 min. After completion of the operation, remifentanil and propofol infusion was discontinued, and 80 mg of sugammadex was intravenously administered after confirming of the return of two twitches with the train of four. We extubated her after confirming that sufficient spontaneous respiration appeared. After extubation, no abnormalities were observed in respiratory and circulatory states. The patient’s postoperative pain was well suppressed with the regular intake of 300-mg acetaminophen. Postoperatively, no anesthesia-related complications were observed, and she was discharged the day after surgery.

## Discussion

HCS is a rare, autosomal dominant disorder, which was first describe by Hajdu and Kauntze in 1948 and subsequently by Cheney in 1965. The main characteristic feature is progressive acro-osteolysis, which results in extremity fractures and deformity of the spine and skull from childhood. Other characteristic features are short stature, dental anomalies, osteoporosis, conductive hearing loss, and vertebral compression [1].

There have been two reports on general anesthesia in patients with HCS managed by orotracheal intubation [2, 3]. One was an 8-year-old girl scheduled for tonsillectomy and adenoidectomy. Preoperative evaluation revealed a short neck, class III Mallampati score, and multiple unerupted teeth. She was intubated using a Miller 1.5 laryngoscope after induction with sevoflurane [2]. The other was a 10-year-old girl with basilar impression, syringomyelia, and obstructive hydrocephalus and scheduled for foramen magnum decompression. Preoperative evaluation revealed dysphagia, sleep apnea syndrome, and aspiration pneumonia. She underwent slow induction with sevoflurane and was intubated using a bronchofiberscope with cervical midline fixation to avoid cervical fracture [3]. Both surgeries proceeded uneventfully, and the patients were extubated postoperatively.

We performed nasotracheal intubation for oral surgery. Preoperative CT scans revealed no skull base fractures or cervical vertebral compression fractures. After induction of general anesthesia, we inserted a suction catheter into the nasal cavity before inserting a nasal airway, which was used as a guide to prevent skull base fracture. We maintained the midline cervical position and the mouth closed during nasotracheal intubation, in order to prevent cervical fractures by retroflexion of the neck, and dislocation of the upset teeth by opening the mouth, in contrast with a previous report of a patient with oral intubation because of the risk of fracture at the skull base [3]. Additionally, since HCS advances with aging and dysmorphic features may worsen over time, the airway should be reassessed carefully before each surgery [7]. HCS seems to be one of the difficult airway-related syndromes, because of the characteristics in skull and neck abnormality [8]. In many HCS cases, micrognathia was presented, and Crifasi et al. reported a HCS case that presented upper airway obstruction [9, 10]. Therefore, the careful preoperative evaluation and tracheal intubation procedure were mandatory for safe airway management, even if the tracheal intubation was performed via oral cavity. Additionally, the fragility of the skull base must be considered in nasotracheal intubation to prevent skull base fracture and cranial nerve disorder [11]. If a skull fragility or fracture is suspected, it is necessary to discuss with the surgeons whether airway management by oral intubation rather than nasotracheal intubation is possible preoperatively.

Our patient also had bilateral renal cysts, but her renal function was normal. However, a patient with HCS may have renal dysfunction due to renal cysts [11]. Renal dysfunction may delay drug excretion and prolong the effect of the drug [12]. Therefore, it is important to evaluate renal function in the preoperative examination of a patient with HCS.

Patients with HCS may also have congenital heart disease such as ASD, ventricular septal defect, and patent ductus arteriosus, and echocardiography is recommended for these patients [13]. Our patient was diagnosed with ASD as an infant but had spontaneous closure at age 8 years. She had a high exercise tolerance of more than 4 metabolic equivalents. The surgery itself was a low-risk procedure, and we determined that the patient could tolerate the general anesthesia well enough. If a patient with congenital heart disease undergoes surgery, we need to manage anesthesia with consideration of the patient’s hemodynamic status.

## Conclusion

We were able to safely manage the airway of a patient with HCS by nasotracheal intubation under general anesthesia. In a patient with HCS, careful preoperative evaluation of the airway and the procedure to prevent skull base damage are necessary for safe airway management with nasotracheal intubation.

## Data Availability

Not applicable.

## Abbreviations

HCS: Hadju-Cheney syndrome
ASD: Atrial septal defect
CT: Computed tomography
